# Ejection and filling rates assessed from cardiac magnetic resonance imaging: possible indices of Degenerative Mitral Valve Regurgitation

**DOI:** 10.1186/1532-429X-18-S1-P347

**Published:** 2016-01-27

**Authors:** Stephanie Marchesseau, Jamie X Ho, John J Totman, Lieng H Ling

**Affiliations:** 1 CIRC, Singapore, Singapore; 2 grid.4280.e0000000121806431Cardiac Department, National University of Singapore, Singapore, Singapore

## Background

Degenerative Mitral Valve Regurgitation (DMR) is a severe form of mitral valve disease for which the survival rate is poor if left untreated. Defining the optimal timing for surgery is challenging in spite of advances in diagnostic imaging. No sensitive measure of left ventricular dysfunction from CMR have yet been reported since the ejection fraction (EF) is usually preserved in DMR patients. However, to assess LV function, standard analysis of the Cine images only focuses on two time-points of the cardiac cycle (end-diastole and end-systole), discarding more than 80 % of the available data. Available cardiac analysis software now allows the analysis of the entire cardiac cycle in a reasonable time, leading therefore to a full LV volume curve over time. The aim of this study is to assess the sensitivity of two indices extracted from the LV volume curve: the peak ejection rate and the peak filling rate.

## Methods

30 patients with clinically identified DMR disease (22 males, mean age 52 range = 26-76) were recruited as well as 10 controls without known heart disease (8 males, mean age 50 range = 32-65). Both groups were scanned on a Siemens Trio 3T MRI. In addition to the localizer, breath-hold CINE steady state free precession (SSFP) short axis sequences were acquired (slice thickness 8 mm with 20% gap). Global LV function was quantified blindly using Segment v1.9 R3556 (http://segment.heiberg.se) by 3 trained analysts. Several indices were extracted from the full LV volume curve: the ejection fraction, the maximum and minimum amplitude of the flow (derivative of the volume with respect to time) also referred to as the peak ejection rate (during systole) |min(dV/dt)| and peak filling rate (during diastole) |max(dV/dt)|. Statistical 2-sample t-test was performed on these 3 indices to measure their sensitivity between the patient group and the control group.

## Results

**Figure 1:**
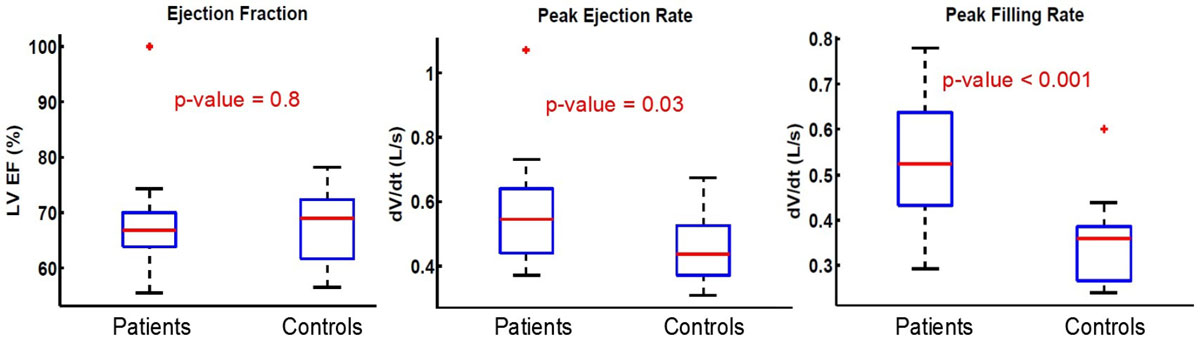
**Statistical comparison of the ejection fraction, peak ejection rate and peak filling rate for the patient and control groups**.

As shown in Figure 1, the ejection fraction shows no significant difference between patient and control groups. However, as already reported in previous studies, patients with DMR present a higher peak ejection rate (|min(dV/dt)| = 0.56 (std 0.14) for patients compared to |min(dV/dt)| = 0.45 (std 0.14) for controls, p-value = 0.03). In addition, in our study, the patient group demonstrates a significantly higher peak filling rate (|max(dV/dt)|= 0.52 (std 0.13) compared to |max(dV/dt)|= 0.35 (std 0.10) for controls, p-value<0.001).

## Conclusions

This study confirms the physiological understanding of DMR. The LV empties rapidly preferably into the low impedance left atrium rather than the high pressure aorta. The ejection fraction being preserved, the LV relaxation must also be faster. In addition, this study shows that two indices currently not measured in CMR could be predictors for degenerative mitral valve regurgitation severity. With now available analysis software, the peak filling rate and the peak ejection rate could easily be measured in a routine clinical environment.

